# Online consultation in an orthopedic trauma surgery outpatient clinic: is there a learning curve?

**DOI:** 10.1186/s12891-022-05144-9

**Published:** 2022-03-02

**Authors:** Pierre Hepp, Georg Osterhoff, Peter Melcher, Ralf Henkelmann, Jan Theopold

**Affiliations:** Department of Orthopedic, Trauma, and Plastic Surgery, University of Leipzig, Liebigstraße 20, D-04103 Leipzig, Germany

**Keywords:** Online consultation, Telemedicine, COVID-19, Clinical examination

## Abstract

**Background:**

In the context of the German contact restrictions due to the COVID-19 pandemic of March 2020, an online-based consultation system was established in our university orthopedic outpatient department to maintain patient care. As a basis for contact-minimizing communication, this was continued after the contact restrictions were lifted. The aim of this prospective pilot study was to assess the effectiveness, technical feasibility, and patient flow in this system under lockdown conditions and in the period afterwards.

**Methods:**

The evaluation took place from the beginning of the first lockdown on March 13, 2020, until May 31, 2021. For each patient encounter, the quality of the sound and video connections was documented. The outcomes of the consultations were recorded. Four categories were distinguished: 1) no follow-up necessary, 2) follow-up via online consultation, 3) referral for surgical therapy, and 4) follow-up in the outpatient clinic for physical examination.

A comparison was made between an early cohort right after implementation of the online consultation and and a late cohort after establishment of the consultation.

**Results:**

There were 408 patient encounters via online consultation. A total of 360 (88%) consultations were uninterrupted. Initial presentations accounted for 124 (30%) consultations. In 75 (18%) patients, no further follow-up was necessary. Follow-up via online consultation was scheduled in 82 (20%) patients, direct referral for surgery was made in 86 (21%) patients, and a follow-up for physical examination was arranged in 165 (40%) patients. When comparing the early and late cohort, there was no difference in the duration of the conversation (*p* = 0.23). A significant difference was found in the type of further treatment. In the late cohort, conservative therapy was used more often (*p* < 0.01), resulting in a lower number of follow-up visits for clinical examination (*p* < 0.01).

**Conclusion:**

While a definite decision for further procedure was possible solely by online consultation in a large percentage of cases, 40% of patients still needed an additional in-person consultation for physical examination. A learning curve could be observed regarding the selection of patients suited for online consultation. Overall, online consultation is a useful measure to manage patient volume and to visibly support direct doctor-patient contact.

## Background

During the COVID-19 pandemic, contact-minimizing measures were adopted in line with the German Infection Protection Act [1–3]. This also enabled hospitals to create capacity for infected patients [3–5]. With the start of the contact restriction, the so-called “lockdown” in March 2020 [3, 6], outpatient treatment options were also increasingly restricted. In accordance with the recommendations of the American College of Surgeons and the German Society for Orthopedics and Trauma Surgery, elective procedures were canceled in order to maintain adequate bed capacity [4, 5, 7]. A reduction in patient volume also became necessary in the university outpatient clinic setting [8]. Online-based formats, or so-called online consultations, offered an alternative.

Encouraged by policy makers, the first attempts to implement digital consultations in surgery were made as early as 2015 [9–11]. Proposals were also made in Germany to integrate digital consultations into everyday practice [12]. Finally, in 2018, the Association of Statutory Health Insurance Physicians set out to promote a digitally based presentation of patients [13]. In this context, online consultation offers flexibility, reduces travel, and prevents direct doctor-patient contact with its increased risk of infection, especially during pandemics [14]. Although a virtual consultation lacks the specific clinical orthopedic tests essential to the examination, an inspection of joint mobility and self-palpation of the joint by the patient are possible [15]. Studies have pointed out the equivalence of virtual and in-person consultations under certain conditions [16, 17]. Immediately after the first lockdown of the COVID-19 pandemic on March 2020, the Department for Arthroscopic and Special Joint Surgery/Sports Injuries of the Clinic for Orthopedics, Trauma Surgery, and Plastic Surgery at Leipzig University Hospital transitioned to an Internet-based online consultation system. This service continued even after the lockdown measures were lifted.

The aim of this prospective pilot study was to assess the effectiveness of an Internet-based consultation in terms of acceptance, technical feasibility, performance, and control of patient flow under lockdown conditions and in the period thereafter.

## Methods

Voluntary online video consultations were conducted for patients from March 13, 2020, until May 31, 2021. Patients were scheduled in advance for online video consultation after telephone appointment coordination. Recognition of the local ethics committee (University of Leipzig) is not necessary; the data collection was based on §34 of the Saxon Hospital Act and performed in accordance to the relevant guidelines and regulations. All patients gave their written informed consent before undergoing the consultation.

A pilot study on the implementation of online consultation with partial results has already been published [18].

Two providers conducted the Internet-based consultations using Sprechstunde.online (Zava Sprechstunde Online GmbH, Essen, Germany) and Samedi.de (Samedi GmbH, Berlin, Germany) software. If there where images, they are sent by the patients via CD to the outpatient clinic in the week before the appointment if there is no VPN direct connection with the radiology offices.

Parallel to the recording of clinical findings in the patient file, a prospective documentation of parameters related to the quality of the online consultation was performed. The duration of the individual consultation was recorded, and the video and audio quality were documented by means of dichotomous questions. Furthermore, the reason for presentation, whether it was a first or a repeat encounter, and the diagnostic and therapeutic outcomes of the online consultation were documented. The outcomes were classified into one of four categories:

1. No follow-up: Follow-up was recommended only if necessary, or further treatment was initiated by a colleague.
2. Online consultation: The patient was referred for a follow-up online consultation.
3. Referral for surgery: Surgery was performed in cases of clear findings and complete imaging. The patient was seen and fully examined in person the day before the operation, and the indication for surgery was confirmed.
4. Follow-up in the outpatient clinic: Referral to the outpatient clinic for more precise clinical evaluation in cases of unclear findings or the need for further imaging.

To investigate the potential presence of a learning curve, patients were divided into an early and a late cohort. The first 100 patients were assigned to the early cohort and the rest to the late cohort. The duration of the consultation and the further treatment categories (1–4) were compared between the cohorts.

For statistical analysis, SPSS (version 24, SPSS Inc., Chicago, IL) was used.

## Results

**Fig. 1:**
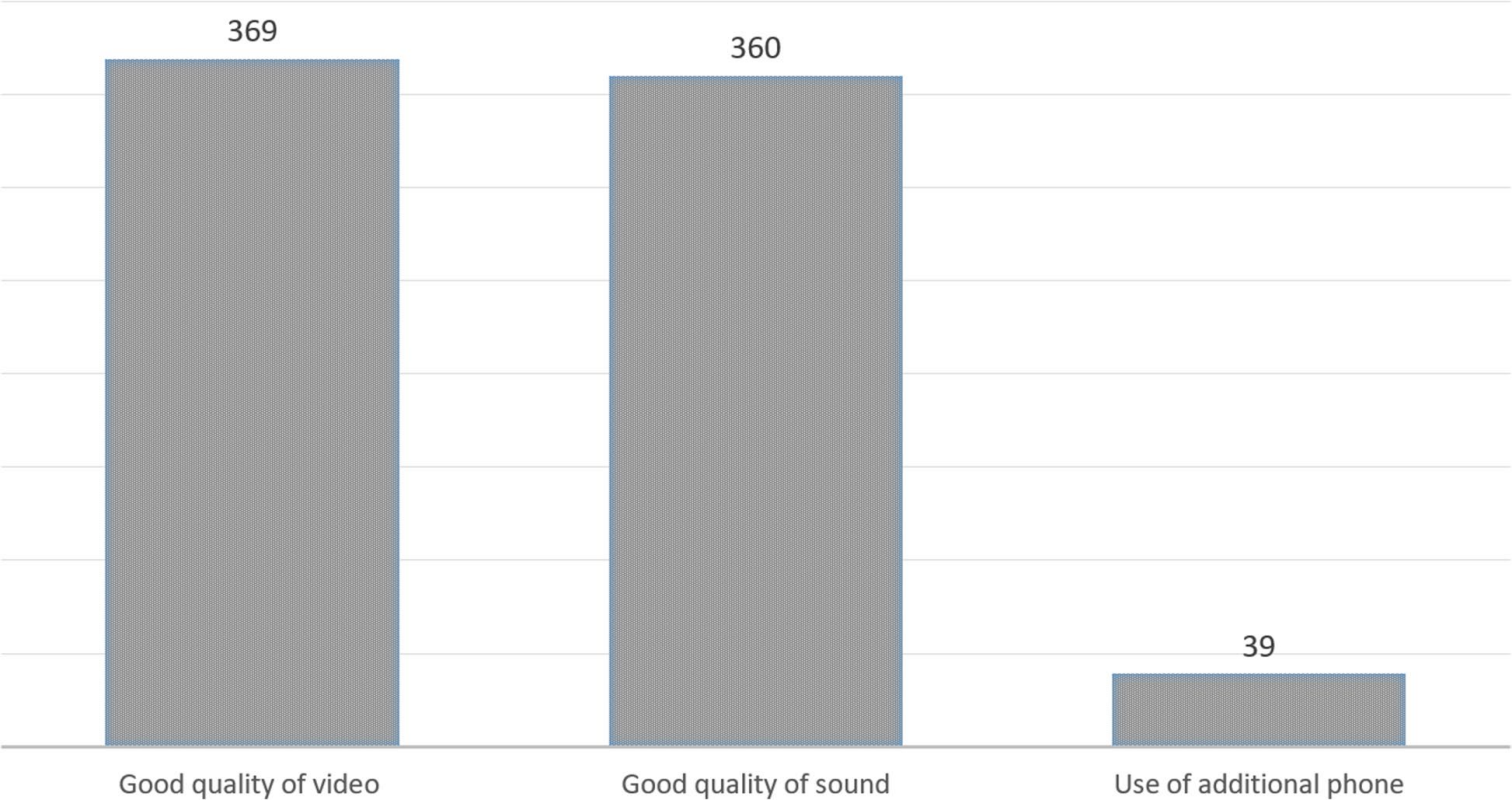
Technical quality of online consultation

A total of 408 Internet-based patient encounters occurred during the study period. The average age of the participants was 39 years (range, 15–83 years), and 175 (43%) were women. Overall, 360 (88%) online video consultations were completed without interruptions (Fig. 1).

**Fig. 2:**
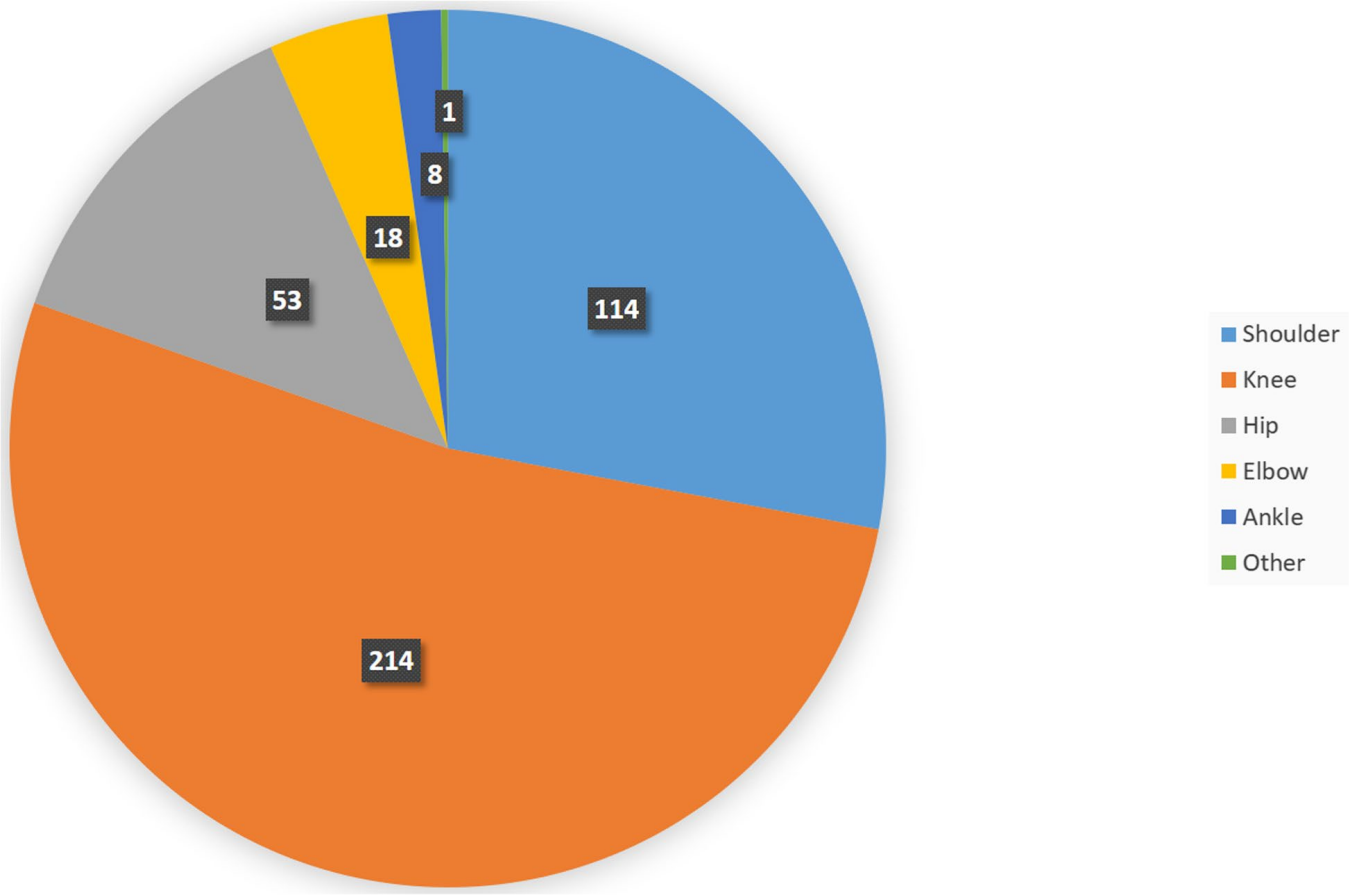
Frequency of involvement of the affected joints/regions

The average in-call time was 9 min 13 s (minimum, 1 min 20 s; maximum, 23 min 25 s). There were limitations in video quality in 31 (10%) contacts and in audio quality in 48 (12%). The average time of conversation in group A was 8.7 min (SD 3.6). In group B, 9.4 min (SD 4.2). A significant difference could not be shown (*p* = 0.24). In 39 (10%) encounters, consultation by telephone was required because Internet-based contact was not possible or the video quality was poor. In all, 124 (30%) encounters were initial consultations, and 284 (70%) were follow-up consultations after inpatient or outpatient treatment. The joint most commonly involved in online encounters was the knee (214 patients, 53%) (Fig. 2). Shoulder pain was the primary symptom in 114 (28%) patients. The hip was involved in 53 (13%) consultations, the elbow in 18 (4%), and the ankle in 8 (2%). Proximal avulsion of the hamstring tendons and peroneal tendon tear were diagnosed in 1 patient each (0.2%).

**Fig. 3:**
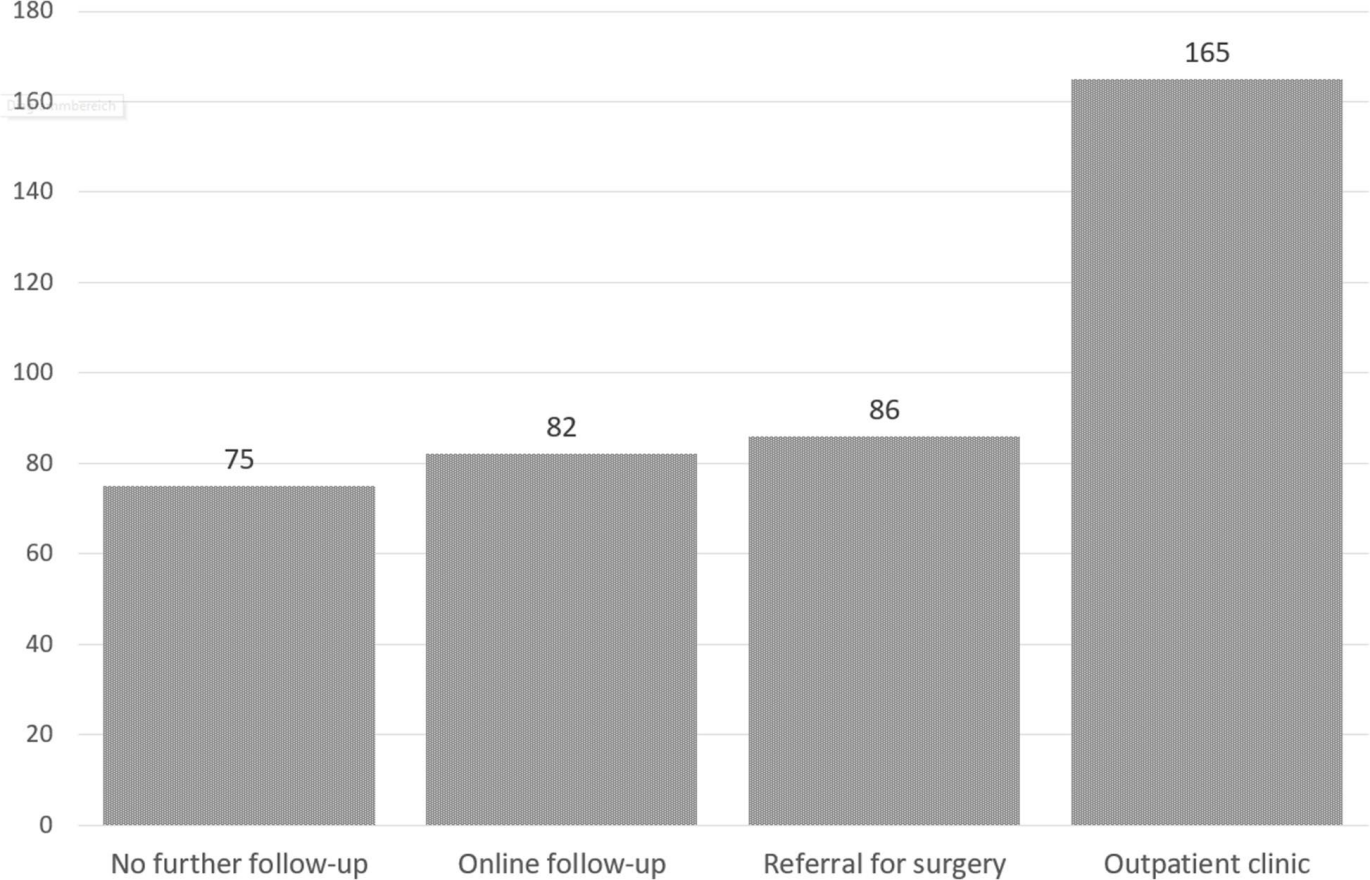
Results of the consultations

**Fig. 4:**
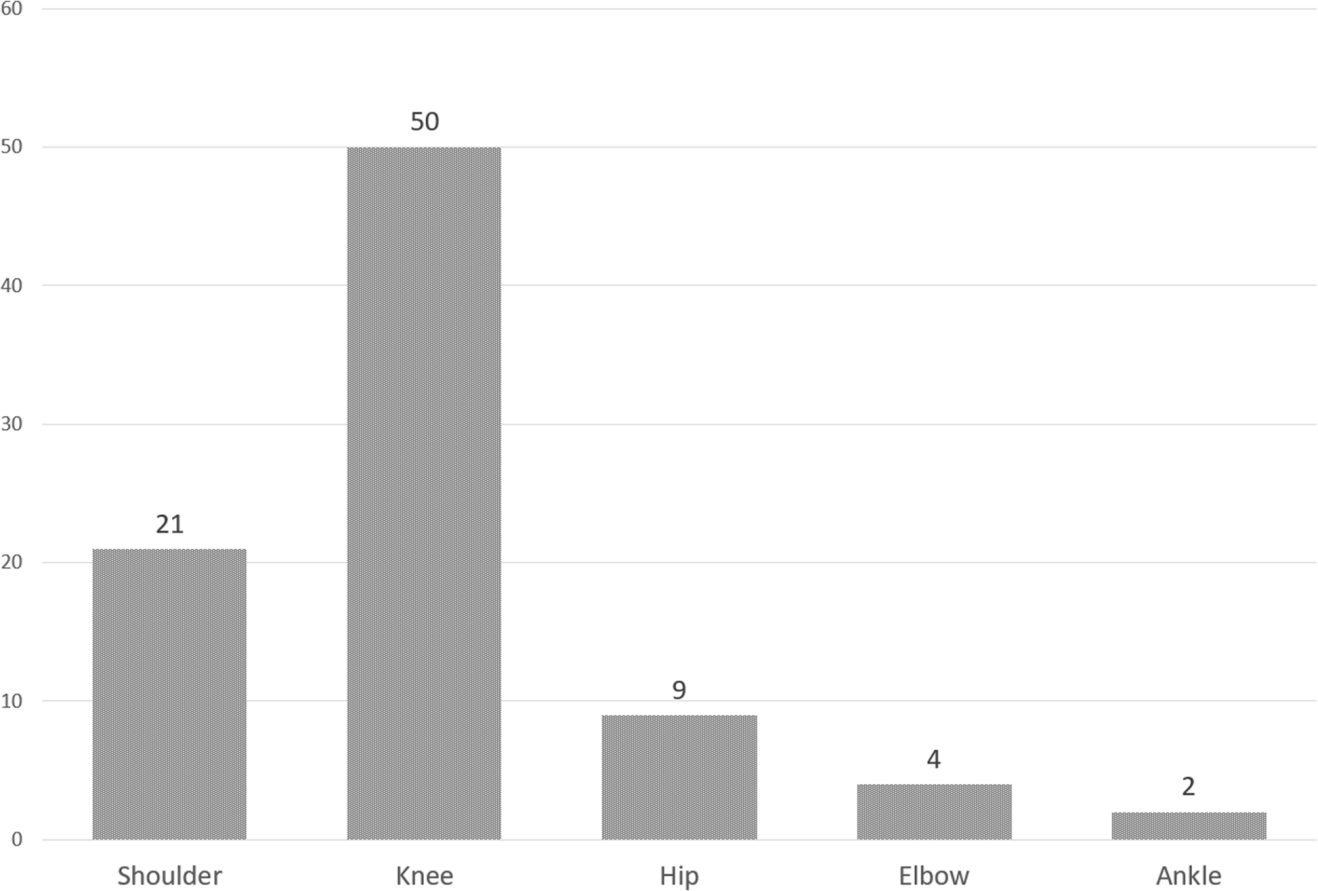
Joints/regions operated on

Regarding consultation outcomes, 75 (18%) patients were referred for a new appointment to our outpatient clinic if necessary or for further treatment by a colleague in private practice (Fig. 3). An online follow-up visit was scheduled in 82 cases (20%). In 165 (40%) patients, follow-up was scheduled for an in-person physical examination and verification of clinical findings assumed during the online consultation. Direct referral for surgery was possible in 86 (21%) patients based on the available resources. Of these, surgery was indicated in 50 (58%) patients for knee problems, in 21 (24%) patients for shoulder complaints, in 9 (10%) patients for hip complaints, in 4 (5%) cases for elbow complaints, and in 2 (2%) patients complained of ankle pain (Fig. 4).

When comparing the early and the late cohort, there was no significant difference in the duration of the conversation (early: 8.7 min SD 3.6; late: 9.4 min, SD 4.2; *p* = 0.23).

**Fig. 5:**
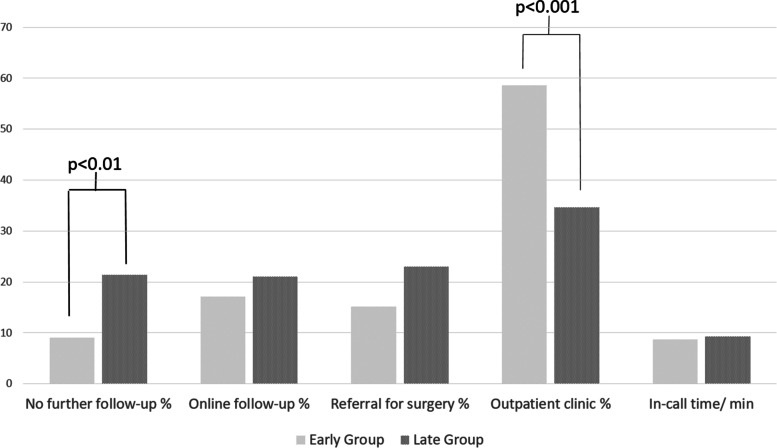
Differences between groups A and B in percentages regarding the further procedure after the consultation in the online consultation hour. On the right, the duration of the consultation in minutes for groups A and B

A significant difference was found in the type of further treatment. The late cohort was significantly more likely to receive conservative therapy (early: *n* = 9 (9%); late: *n* = 66 (21%); *p* < 0.01) and significantly less likely to return for an in-person clinical follow-up (early: *n* = 58 (58%); late: *n* = 107 (34%); *p* < 0.01). No difference could be found with regard to the further treatment” surgery” (early: *n* = 15 (15%); late: *n* = 71 (23%); *p* = 0.12) and “online follow-up visit” (early: *n* = 17 (17%); late: *n* = 65 (21%); *p* = 0.4) (Fig. 5).

## Discussion

In Germany, the number of doctor visits has increasingly been critically discussed [19]. On average, each patient has ten physician encounters per year [20]. In Sweden, e-health solutions are increasingly used to reduce the number of doctor visits [20]. In the USA, special questionnaires were developed for orthopedic consultations to examine patients during the Covid-19 pandemic [21–23]. In Germany, the availability of online consultations increased only slightly in the beginning of the pandemic [24]. As the pandemic progressed, protocols and investigative techniques were increasingly developed and evaluated in Germany as well [25, 26]. Buvik et al. showed that online consultations were considered equivalent to in-person consultations by Norwegian orthopedic surgeons [10, 27].

Our patient sample showed good acceptance of services such as online consultation. The collective of the consultations included younger patients with an average age of 39 (median 37) years who were well-versed and open-minded in the use of modern techniques. However, even older patients are not closed-minded to the integration of modern communication [17]. Thus, this study showed that older patients are also interested in digital consultation.

The average treatment time (patient-doctor conversation) of 9 min 13 s was similar to that of a German general practitioner [28, 29]. It should be noted that neither preparation nor follow-up, including documentation, was considered. Overall, at least for the physician, a reduction in the time spent per patient cannot be assumed.

Our data shows that the duration of the call itself did not change along the learning curve.

A negative aspect is the current lack of regulations regarding the assumption of costs, especially for university outpatient clinics [30]. This concerns, among other things, the issuing of prescriptions for conservative therapy.

Because of improved network coverage, a sufficient network speed is available under present conditions to perform an Internet-based consultation [31]. Even with poor image quality, in some cases, further consultation was possible using only audio. Only in cases of poor sound quality was it necessary to resort to a telephone consultation.

The patient was seen for the first time in 30% of the consultations. In particular, ancillary data such as cross-sectional imaging or consultative examinations (e.g., nerve conduction study/electromyography) can be performed for completeness if indicated. This explains the high number of follow-up outpatient encounters. A significant advantage of online consultation is that the number of patients with incomplete diagnostics can be minimized in a real consultation, thus avoiding multiple encounters.

In 40% of the patients, a definitive decision on further treatment could be made in the online consultation. Conservative functional therapy was initiated, or surgery was planned as indicated.

In terms of learning curve, significantly more patients in the early cohort required no further follow up. This may be due to better selection of patients for the online consultation. On the other hand, there were fewer patients in the late cohort who needed an in-person clinical examination. No difference was found in the frequency of indication for surgery. This seems understandable, since only a limited number of diagnoses can be made accurately enough to justify direct referral for surgery without a thorough clinical examination.

Assessment of the image and sound quality was performed only on the basis of dichotomous questions. A specific quality evaluation is not possible. However, we can conclude that with a stable network connection, the quality of the consultation is sufficient to perform an adequate history. Furthermore, it should be noted that because of the anonymized data collection, multiple presentations of individual patients cannot be excluded.

Online service providers are constantly trying to improve their platforms. The integration of calendars for organizing consultation hours, on the one hand, and the option for patients to choose their own appointments, on the other, are already innovations that make the applications much easier to use. Functions such as screen splitting support the doctor in explaining findings.

What is missing is a good and practical function for the patient to provide his MRI/CT data without complications (e.g. integrated image viewer) for the appointment.

## Conclusion

Online video consultation in the COVID-19 pandemic lockdown and in the period thereafter was shown to be effective in terms of technical feasibility and performance. The time spent by the physicians was not reduced. Patient volume can be controlled to the extent that multiple presentations due to missing preliminary findings and routine control examinations can be reduced. Online consultation can therefore be regarded as a supplementary measure that hardly replaces direct doctor-patient contact with clinical examination.

A learning curve could only be observed regarding the selection of patients suited for online consultation.

Due to the positive results and the high patient acceptance of online consultation, especially from more distant regions, online consultation will continue to be offered in our clinic even after the pandemic.

## Data Availability

All data generated or analysed during this study are included in this published article.
